# Relationship between Autism Spectrum Disorder and Pesticides: A Systematic Review of Human and Preclinical Models

**DOI:** 10.3390/ijerph18105190

**Published:** 2021-05-13

**Authors:** Judit Biosca-Brull, Cristian Pérez-Fernández, Santiago Mora, Beatriz Carrillo, Helena Pinos, Nelida Maria Conejo, Paloma Collado, Jorge L. Arias, Fernando Martín-Sánchez, Fernando Sánchez-Santed, Maria Teresa Colomina

**Affiliations:** 1 Department of Psychology, Research Center for Behavior Assessment (CRAMC), Universitat Rovira i Virgili, 43007 Tarragona, Spain; judit.biosca@urv.cat; 2 Research in Neurobehavior, Health (NEUROLAB), Universitat Rovira i Virgili, 43007 Tarragona, Spain; 3 Department of Psychology, Health Research Center (CEINSA), Almeria University, 04120 Almeria, Spain; cpf603@ual.es (C.P.-F.); santiagomorap@gmail.com (S.M.); 4 Department of Psychobiology, University Institute of Research-UNED-Institute of Health Carlos III (IMIENS), National Distance Education University (UNED), 28015 Madrid, Spain; bcarrillo@psi.uned.es (B.C.); hpinos@psi.uned.es (H.P.); pcollado@psi.uned.es (P.C.); 5 Laboratory of Neuroscience, Department of Psychology, Instituto de Neurociencias del Principado de Asturias (INEUROPA), University of Oviedo, 33011 Oviedo, Spain; conejonelida@uniovi.es (N.M.C.); jarias@uniovi.es (J.L.A.); 6 National Scholl of Public Health, Institute of Health Carlos III, University Institute of Research-UNED-Institute of Health Carlos III (IMIENS), 28029 Madrid, Spain; fmartin@isciii.es

**Keywords:** autism spectrum disorder, sociability, pesticide, organophosphate, carbamates, organochlorine, chlorpyrifos

## Abstract

Autism spectrum disorder (ASD) is a complex set of neurodevelopmental pathologies characterized by impoverished social and communicative abilities and stereotyped behaviors. Although its genetic basis is unquestionable, the involvement of environmental factors such as exposure to pesticides has also been proposed. Despite the systematic analyses of this relationship in humans, there are no specific reviews including both human and preclinical models. The present systematic review summarizes, analyzes, and discusses recent advances in preclinical and epidemiological studies. We included 45 human and 16 preclinical studies. These studies focused on Organophosphates (OP), Organochlorine (OC), Pyrethroid (PT), Neonicotinoid (NN), Carbamate (CM), and mixed exposures. Preclinical studies, where the OP Chlorpyrifos (CPF) compound is the one most studied, pointed to an association between gestational exposure and increased ASD-like behaviors, although the data are inconclusive with regard to other ages or pesticides. Studies in humans focused on prenatal exposure to OP and OC agents, and report cognitive and behavioral alterations related to ASD symptomatology. The results of both suggest that gestational exposure to certain OP agents could be linked to the clinical signs of ASD. Future experimental studies should focus on extending the analysis of ASD-like behaviors in preclinical models and include exposure patterns similar to those observed in human studies.

## 1. Introduction

Autism spectrum disorders (ASD) are a heterogeneous group of neurodevelopmental disorders characterized by varying degrees of altered sociability, reduced communicative skills, and repetitive stereotyped behaviors unfocused on a specific goal [1]. Although these constitute the core clinical signs of ASD, it is well-known that ASD-diagnosed children also display alterations in other physical and cognitive functions such as motricity [2], attention [3], memory [4], and inhibitory control [5], amongst others.

Although the etiopathology of ASD remains unknown, there is a consensus regarding the relevance of its heritable/genetic basis, confirmed in various human twin studies [6] and animal models that show a simplified ASD-like phenotype, as in the case of the fragile X mental retardation (FMR1)-KO rodents, the BTBR models, and the neuroligin 3 (Nlgn3)-KO rodents, amongst others [7]. There is considerable empirical support for this genetic component [8], with multiple genes and loci found to be strongly associated with the diagnosis of ASD, of which the matrix metallopeptidase 12, neurotrimin, potassium calcium-activated channel subfamily *N* member 2, and the microtubule-associated protein tau are particularly noteworthy. However, the prevalence of ASD has increased significantly in recent decades [9], which can presumably be taken to indicate that heritability and genetic background are not the only causes of this set of disorders. This impressive rise in the prevalence of ASD diagnoses can be partially explained by changes in diagnostic criteria (for a good example, see [10]).

Although the impact of these changes is unquestionable, this factor is not able to explain completely this sharp rise in diagnoses. Other authors have proposed that the incidence of external factors could interact with the developmental central nervous system (CNS) by inducing physiological and functional abnormalities linked to the development of ASD and/or its degree of severity [11]. In general, the etiology of ASD is considered from a multifaceted perspective, where the relevant genes play an important role as vulnerability factors that are modulated by external environmental variables influencing the course and severity of the final behavioral and molecular outputs observed in the diagnosed patients [12]. Some of the most significant environmental factors are perinatal stressors, infections, socioeconomic status, dysfunctional familiar relations, and exposure to specific agents with a neurotoxic profile [11,12]. From the latter category, the most important toxic compounds linked to ASD are heavy metals, persistent organic pollutants, and valproic acid (VPA) [13], along with exposure to pesticides [14,15,16]. These aspects make the study of ASDs particularly suitable to new research approaches such as those included in exposome science [17]. Developments in exposome science have revealed the need for evaluating the interrelationships among phenotype, genotype, and exposure data (expotype).

Concerning neurotoxic agents, various families of pesticides have recently been linked with ASD. Of these, Organochlorine (OC), Pyrethroids (PT), Carbamates (CM), Neonicotinoids (NN), and Organophosphates (OP) have been studied in most depth. Briefly, OC compounds are synthetic pesticides widely used globally, with important applications in both industry and agriculture and whose main mechanisms of toxicity are the regulation of the axonal sodium gates (DDT-Type) and GABAergic regulation of Chloride ion influxes (Chlorinated Alicyclic-type) [18]. Some of the most important OC agents are DDT, DDD, Eldrin, Dieldrin, and Endosulfan, amongst others [19]. Moreover, PT agents are compounds commonly used as insecticides that induce excitatory paralysis by directly acting on the voltage-gated sodium channels [20]. Some of the most representative PT compounds are Allethrin, Dimethrin, Tetramethin, and Alphametrin [19]. CM xenobiotic compounds generally induce their toxic profile by reversibly inhibiting the Acetylcholinesterases (AChE), thus increasing the cholinergic tone in the CNS [21]. From this chemical family, Carbaryl, Aldicarb, Pyrolan, and Carbanolate are among the most notable methyl agents [19]. Added to this, NN xenobiotic agents are a group of effective insecticides whose main use is focused on the control of sucking insects and flea control, since they act as selective agonists of nicotinic receptors [22]. Of these, Imidacloprid, Thiamethoxam, and Clothianidin are among the most used [22]. Finally, OP compounds are a wide-range group of pesticides commonly applied in industry, agriculture, and, to a less extent, used for residential purposes [23]. Their main mechanism of toxicity is the irreversible inhibition of the AChE in the CNS. Some of the most noteworthy OP agents are Chlorpyrifos (CPF), Malathion (MAL), Parathion, and Tricholorfan [19].

Both human and rodent studies have found interesting links between exposure to these various pesticides and ASD diagnosis or ASD-like behaviors [24,25]. However, results differ depending on the agent, the dose, the time of exposure, age of behavioral assessment, and the outcomes measured. Thus, whilst it appears that we have sufficient empirical results to establish solid conclusions, this is not actually the case, since the different studies have not been adequately analyzed based on the specific characteristics previously defined. Moreover, there is a lack of adequate discussion regarding the quality of each individual study.

Given these considerations, we thought it worthwhile to conduct a systematic review of the most important empirical studies concerning exposure to different pesticides and their effect on ASD diagnosis and core behaviors in humans, and on ASD-like outcomes in pre-clinical rodent models. In doing so, the scientific community will have access to a clearer picture of the real relationship between exposure to these compounds and the incidence of ASD.

## 2. Materials and Methods

### 2.1. Review Protocol

Prior to the literature search and in accordance with the ‘’Preferred Reporting Items for Systematic Review and Meta-Analysis Protocol’’ (PRISMA-P) Moher et al. [26] a detailed review protocol was created. As recommended by the PRISMA-P guidelines, and according to the suggestions of the PROSPERO reviewers, two registrations were created on the PROSPERO database: one for animals (Prospero-ID: 145135, October 2019) and one for humans (Prospero ID: 153081, October 2019).

### 2.2. Eligibility Criteria

The systematic review was structured initially with the aid of the PICOS acronym (Participants, Interventions, Comparators, Outcomes measures, Study design). Participants were young humans (children or adolescents) and rodents. Interventions were prenatal or postnatal exposures to potential neurotoxic pesticides, herbicides, or insecticides. Moreover, comparison of environmental exposure (pre or postnatal) to pesticides, herbicides, or insecticides with a control/non-exposed group or comparison between groups with different levels of exposure (i.e., low, medium, high) were assessed according to the proximity to agricultural/industrial areas or the metabolite levels in blood/urine samples.

Biological outcomes such as agent exposure biomarkers (metabolite levels in blood or urine samples), hormonal alterations (enzymatic analyses in blood or urine), neurotransmitter activity alterations (protein levels in immunochemical arrays of blood and tissue samples and genetic expression in PCR), and cytokine alterations (protein levels in immunochemical arrays of blood and tissue samples) were assessed. Furthermore, behavioral measures related to autism such as cognitive and psychomotor alterations, as well as social communication impairments, were also evaluated.

In addition, experimental studies in the literature with animal models and cohort, cross-sectional and case-control human studies were considered, along with studies written in English and published within the last ten years. Exclusion criteria were defined by considering those aspects that did not meet the previously defined PICOS characteristics. Therefore, we did not select case studies, reviews, abstracts, or communications at scientific meetings, or qualitative studies. Lastly, we only included articles published in peer-reviewed journals.

### 2.3. Information Sources

We carried out comprehensive literature searches of Pubmed and Scopus until March 2020. The keywords used were autism spectrum disorder (ASD), pesticides, insecticides, herbicides, gestational, prenatal, and postnatal exposure, neurodevelopment, humans (plus combinations). Filters employed in the database searches were language (English) and data publication (last ten years). The search formula was: (TITLE-ABS-KEY (pesticides) AND (TITLE-ABS-KEY (prenatal AND expos*) OR TITLE-ABS-KEY (postnatal AND expos*) OR TITLE-ABS-KEY (gestational AND expos*) AND TITLE-ABS-KEY (autism*) OR TITLE-ABS-KEY (ASD) OR TITLE-ABS-KEY (neurodevelopment*). In Scopus, an asterisk served as a substitute for any number of characters, expanding the search. Furthermore, a hand-search was performed in relevant journals and the reference lists of reviews focusing on the subject.

### 2.4. Study Selection and Data Collection Process

After eliminating duplicates, one reviewer examined the complete list of results for eligibility. If further relevant decisions were to be made, these were discussed among the research team until reaching a consensus. Moreover, two reviewers independently extracted the data in an unblended manner. Any disagreements were resolved until achieving consensus.

### 2.5. Risk of Bias in Individual Studies

Two tools were employed to assess the risk of bias: for animal studies, we used the ‘’SYRCLE’s tool for assessing the risk of bias’’ [27], which is an adaptation for animal studies based on the Cochrane collaborations RoB tool [28], whilst for human studies, we employed the Newcastle-Ottawa Scale (NOS) to rate cohort and case-control studies [29].

In this regard, the SYRCLE consists of five quality parameters: selection, performance, detection, attrition, and reporting bias. It assigns a maximum of six points for selection, four points for performance, four points for detection, four points for attrition, and four points for reporting (for a total of 18 points). Therefore, the total quality index score was ranked as follows: 0 to 3, 4 to 6, 7 to 9, 10 to 12, 13 to 15, and 16 to 18, these being very low (VL), low (L), medium-low (ML), medium-high (MH), high (H), and very high (VH) quality, respectively. For human studies, the NOS uses three quality parameters: selection, comparability, and exposure/outcome assessment. It assigns a maximum of four points for selection, two points for comparability, and three points for exposure or outcome (making a total of 9 points). Hence, the total quality index score was ranked as follows: 0 to 2, 3 to 4, 5 to 6, and 7 to 9, these being L, MH, H, and VH quality, respectively.

## 3. Results

### 3.1. Selection of Studies

**Figure 1:**
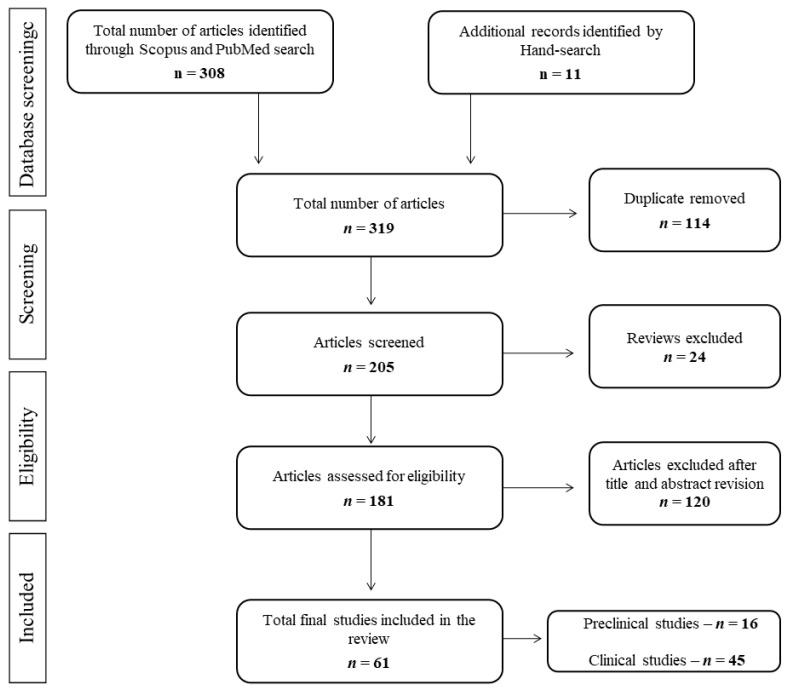
Flow Diagram. From top to bottom, the total number of outputs from Scopus and PubMed searches (*n* = 464). Selection based on year, language (*n* = 308) and exclusion of duplicates (*n* = 194), the total number of studies that successfully passed the selection checklist (*n* = 50), and the total number of accepted studies after parallel searching (*n* = 61).

A flow diagram illustrates the whole search strategy (Figure 1). The first screening provided a total of 464 studies, and after removing duplicates and selecting articles based on year, language, and exclusion of reviews, a total number of 170 articles were selected. Further, 7 preclinical and 43 clinical studies were eliminated after reviewing the title and abstract. A parallel search based on words mentioned previously allowed us to find a further nine preclinical and two clinical studies. Thus, 16 preclinical and 45 clinical studies comprised the total number of studies included in this review.

### 3.2. Search Results and General Quality

Sixteen preclinical and 45 clinical studies were accepted. With regard to the studies conducted in rodents, the majority exposed the animals to CPF or CPO (11 out of 16, 68.5e%) [30,31,32,33,34,35,36,37,38,39,40]. A further two studies used other OP compounds such as CPF, MAL [41], and Phosphomedon (PMD) [42]. That is, most of the studies included in the present review are concerned with OP exposure (13 out of 16, 81.3%). Of the remaining articles, two used Glufosinate ammonium (GLA) (12.5%) [43,44], and one exposed their animals to the synthetic Pyrethroid cypermethrin (CYP) (6.3%) [45]. From all of these studies, only one [42] did not administer the pesticide during development; thus, most of the studies included in this systematic review present good examples of developmental neurotoxicity (15 out of 16, 93.8%). Interestingly, 10 out of the 16 studies (62.5%) followed a gestational exposure protocol, two used postnatal exposure (12.5%), and three employed a continuous exposure regime during both gestational and postnatal stages (18.8%). All of these studies, with the exception of the adult study, used mice models. Basaure et al. [40] also exposed their rodents to CPF during adulthood along with postnatal exposure.

Regarding the studies conducted in humans, 17 out of 45 (37.8%) studied OP exposure using urine, blood, and house-dust metabolite measures [46,47,48,49,50,51,52,53,54,55,56,57,58,59,60,61,62], with OP being the most studied pesticide in this review. From the remaining articles, 9 out of 45 (20.0%) studied OC exposure by DDT and/or DDE exposure [63,64,65,66,67,68,69,70,71], while Puertas et al. [72] and Boucher et al. [73] used other OC compounds such as Mirex and Chlordecone, respectively. Thus 11 out of 45 (24.4%) of the total studies were related to OC exposure. Further, PT exposure was assessed in 3 out of 45 studies (6.7%) [74,75,76], whereas exposure to Permethrin, and Piperonyl butoxide (PBO) (a synergistic component of pesticide formulation) and the metabolite of PT insecticide 3-phenoxybenzoic acid (3-PBA) exposure were reported by Horton et al. [77] and Watkins et al. [78], being five (11.1%) the total PT studies. Additionally, Zhang et al. [79] and Mora et al. [80] used Carbofuranphenol and Mancozeb compounds to assess CM exposure, whereas Keil et al. [81] assessed Imidacloprid exposure, a NN pesticide. Finally, 9 out of 45 (20.0%) studies [82,83,84,85,86,87,88,89,90] reported exposure to a common mix of different types of pesticides. From all these studies, 37 out of 45 (82.2%) followed a longitudinal design, while only 8 (17.8%) were case-control studies. Moreover, 32 out of 45 (71.1%) defined a prenatal exposure period, two (4.4%) a postnatal exposure period, while the remaining 11 (24.4%) considered both prenatal and postnatal periods. In human studies, exposure outcomes were assessed during childhood or adolescence.

Concerning the quality of the preclinical studies, three out of the 16 were labeled as H (18.8%) [30,37,38], 10 as MH (62.5%) [31,32,33,34,35,36,40,43,44,45], and the remaining three as ML-quality (18.8%) [39,41,42]. In a similar vein, 37 out of 45 human studies were classified as VH (82.2%) [46,48,49,50,51,52,53,56,57,58,59,61,63,64,65,66,67,70,71,72,73,74,75,76,77,80,81,82,83,84,85,87,88,89,90,91], 7 as H (15.6%) [47,54,55,60,68,69,78] and only Woskie et al. [62] was classified as MH-quality.

### 3.3. Pesticide Exposure and ASD-Like Outcomes: Preclinical Studies

All of the preclinical studies concerned with pesticide exposure and ASD-like behaviors are summarized in Table 1.

#### 3.3.1. Organophosphates Compounds

There were no preclinical studies labeled as VL or L-quality. Three out of the 13 OP studies were labeled as ML (23%) [39,41,42], seven were labeled as MH (54%) [31,32,33,34,35,36,40] and only three studies were classified as H-quality (23%) [30,37,38]. The OP studies were characterized as following a gestational exposure protocol (eight out of 13, 62%), although some studies used postnatal (2 out of 13, 15%) [30,40] and continuous gestational-postnatal exposure protocols (two out of 13, 15%) [33,41], with the exception of the adult study. Four of these studies used different genetic models of autism such as BTBR mice [35,36] and KO reeler mice [39] and other genes that could potentially modulate social behavior such as the different polymorphism of the human Apolipoprotein E (APOE) [40].

Gestational exposure to OPs both decreased [31,37,38] and enhanced [32,34,35] different social and communicative behaviors in mice. Lan et al. [37] exposed male mice from GD12 to 15 using a dose ranging from 2.5 to 5 mg/kg/day and found decreased social interaction and altered preference in a socially conditioned paradigm, without maternal care alterations. This result was confirmed in a later study [38] in both exposed males (highest dose) and females (lowest dose), without observing significant effects of the exposure on hypothalamic oxytocin mRNA expression. Further, Venerosi et al. [31] found that these abnormal social interaction patterns were complemented by alterations in communication skills, observing a decrease in the number and duration of the exposed pups’ ultrasonic vocalizations (USVs) as well as alterations in maternal behavior (increased licking and exploration-tendency) following 6 mg/kg of CPF from GD15 to 18. Venerosi et al. [32] found a decrease in maternal aggressive behavior, increased anxiety in females, and a generally hyposensitized serotonergic system. De Felice et al. [34] exposed their mice following a similar exposure protocol and found that the exposed females showed enhanced social investigation rates. Further, De Felice et al. [35] also found an increased rate of USVs and social investigation in males using the same exposure protocol in BTBR mice. In addition to their previous observations, De Felice et al. [36] found that the BTBR mice had, at baseline, higher levels of two of the most significant biomarkers of oxidative stress, 15-F2t-IsoP, and PGE2, both of which are associated with ASD. Interestingly, these authors found that this gestational exposure to CPF generally increased the levels of these molecules, eminently in male BTBR mice, which is congruent with their previous findings of altered neuromotor development in BTBR exposed animals [35]. Finally, Mullen et al. [39] used heterozygous/homozygous reeler mice (*Rl*^+/−^ and Rl^+/+^, respectively) and exposed them to CPO (6 mg/kg/day) from GD13 to delivery. The authors found that the exposure increased the number of USVs in the +/− male mice, but the opposite was found in the +/+ condition, whilst the females decreased USVs showed increased levels of social interaction in both genetic conditions.

Postnatal exposure to OPs was analyzed in Venerosi et al. [30] and Basaure et al. [40], both studies using CPF at the preweaning developmental stage. Venerosi et al. [30] found that 3 mg/kg from PND11 to 14 had no effects on the sociability indexes of mice but altered maternal care and social investigation whilst also reducing maternal aggression, a finding that was presumably related to a decreased state of anxiety in the exposed female rats. Further, Basaure et al. [40] used human APOE-3 or 4 mice models to characterize the presumable influences of genetic background on the social mismatches associated with preweaning CPF exposure (1 mg/kg/day from PND10 to 15). They also repeated this exposure during adulthood in both postnatal exposed and non-exposed groups. Postnatal CPF exposure increased the reaction to social novelty in APOE4 mice but reduced this reaction in APOE3 mice, this latter effect being blocked following the adult exposure protocol. The authors found that the adult exposure regime enhanced sociability regardless of the prior exposure condition in the APOE3 mice. Furthermore, the authors also analyzed various molecular markers and found that adult CPF exposure differentially modulated the hypothalamic levels of Oxytocin and Vasopressin mRNA (amongst others) depending on genotype background. Interestingly, chronic exposure (30, 45, or 60 days) to PMD (35 ppm) during adult ages did not alter sociability but produced a significant increase in locomotor activity along with multiple histological alterations in rats [42].

Finally, the studies that employed continuous gestational-postnatal exposure were those of Venerosi et al. [33] and Ouardi et al. [41], which used CPF and MAL, respectively. Venerosi et al. [33] exposed mice to 6 mg/kg of CPF from GD15 to PND14 and found a generalized enhancement in social investigation/recognition in both sexes (stronger in males) without altering motricity. Interestingly, the authors also analyzed the levels of oxytocin and vasopressin1a receptor in the amygdala and found that CPF exposure reduced the former in males and increased the latter. Ouardi et al. [41] exposed their mice to MAL (5–15 mg/kg) from GD6 to PND21 and found a significant reduction in sociability and reaction to social novelty in the exposed animals compared with their control counterparts, along with an increased anxiety state and multiple molecular alterations in the CNS such as increased MDA levels and decreased CAT, SOD, GST and GNX in a dose and age-dependent fashion, presumably indicating an increased state of cellular oxidative stress in exposed rodents.

#### 3.3.2. Other Potential Neurotoxic Compounds

All three studies that used xenobiotic compounds other than OPs were labeled as MH-quality [43,44,45]. Dong et al. [44] exposed female mice to GLA from 8 weeks before mating to delivery, whilst Laugeray et al. 43], and Laugeray et al. [45] used GLA (the former) and CYP (the latter) in a continuous gestational-postnatal exposure protocol in mice models. Dong et al. [44] exposed mice to 12 µg/mL of GLA (in water) and found a general decrease in social interaction and reaction to social novelty, along with decreased locomotor activity, increased compulsive/anxiety-like behavior, and a reduction in the mRNA expression levels of the cortical Nrxn1 gene. The authors also found a significant gut dysbiosis characterized by an increase and decrease of both Bacteroidetes and Firmicutes bacteria at the phylum taxa level and reduced biosynthesis of fatty acids, amongst other molecular changes. These effects on sociability were also found in Laugeray et al. [43], who exposed rodents to GLA from early gestational ages to PND14 in a range of doses from 0.2 to 1 mg/kg/day, finding a significant decrease in USVs and social interaction rates following the highest dose (without affecting anxiety-state levels), along with several molecular outcomes such as a reduction in both Pten and Peg3 brain genes, which are commonly associated with ASD. Similarly, Laugeray et al. [45] also found decreased sociability and self-grooming and increased motricity in mice exposed to 5 mg/kg/day of CYP, as well as altered maternal behaviors following a higher dosage (20 mg/kg/day), without effects on USVs and anxiety. Interestingly, the authors also found that these exposure regimens altered multiple genes.

### 3.4. Pesticide Exposure, Cognitive and Behavioral Alteration Related to ASD: Clinical Studies

The clinical studies included in this review evaluated different aspects related to neurodevelopmental, behavioral, and cognitive outcomes. This broad spectrum of study designs, methods, and functions evaluated added certain difficulties since each study assessed different aspects at different ages, thus hindering the possibility of drawing firm conclusions.

The 45 human studies included in this review are summarized in Table 2 according to the quality index. We have further categorized these studies by describing them according to the type of pesticide and period of exposure.

#### 3.4.1. Organophosphate Compounds

We found 17 studies (37.8%) that referred to the association between OP and autism or developmental disorders. Of these, 12 were classified as VH (70.6%) [46,48,49,50,51,52,53,56,57,58,59,61], four as H (23.5%) [47,54,55,60] and only one [62] was classified as MH-quality. Twelve studies out of 17 assessed only prenatal exposure to OP, whilst five out of 17 (29.4%) [46,48,50,57,61] evaluated both prenatal and postnatal exposure and only Gonzalez-Alzaga et al. [55] studied postnatal exposure alone.

All of these studies assessed prenatal exposure to OPs by means of maternal urine or cord blood biomarkers, or child urine biomarkers (in the case of postnatal exposure). In general, the studies analyzed a set of different dialkyl phosphate metabolites (DAP) including dimethyl (DM) phosphate and diethyl (DE) phosphate metabolite [47,48,49,50,51,52,53,54,56,57,58,59,60,61,62]. Only two studies used other metabolites such as 3,5,6-trichloro-2-pyridinol (TCPy) as an indicator of exposure to CPF [46] or a direct estimation of pesticide exposure together with metabolites [60].

Two of these studies, conducted in Thailand and China, evaluated the effects of prenatal exposure to OPs during the first postnatal week [49,62]. In a pilot study, Woskie et al. [62] found a significant positive relationship between maternal urinary DM phosphate metabolites levels and the Bazelton Neonatal Behavioral Assessment (NBAS) habituation cluster score, along with a significant positive relationship between total DE phosphate metabolites and the NBAS range of state cluster score. However, Zhang and coworkers [49] reported a consistent negative association between neurodevelopmental scores and OPs metabolites in maternal urine, that is, they found an association between DEs and lower scores on the behavior scale whilst DMs concentrations were associated with poorer scores in passive tone, active tone, and primary reflex [49].

Another two studies evaluated children during the first year of life, between 6 weeks and 9 months of age [60] and at 5 months of age [56]. The former study measured metabolites and pesticides in cord blood samples from a Chinese population, and whilst they found no effects in six-week-old children, a significant negative association was found between exposure to Naled and CPF and motor function among girls aged nine months [60]. Moreover, Kongtip and coworkers [56] studied a population from Thailand and found an association between concentration of DEs in maternal urine during the third trimester of gestation and a decrease in cognitive and motor function at 5 months of age, as well as a significant relationship between prenatal total DAP levels and motor scores.

In addition, three studies evaluated children aged from one to two years [48] and at two years of age [53,57]. Wang and coworkers [48], studying a population from China, found a significant negative association between prenatal urine levels of DEs and DAPs and social scores (among boys) at two years of age. In addition, in this study, postnatal urine levels of DAPs and DMs were also associated with increased scores on the adaptive domain in children at two years of age [48]. A study conducted in California, with the CHAMACOS cohort, found that maternal DAPs were negatively associated with cognitive and mental abilities as well as with child PON1 polymorphism, whilst no sex differences were reported [53]. Moreover, in a study of a cohort living in Shenyang (China), Liu et al. [57] reported that prenatal DEs levels were associated with an increased risk of developmental delay (in boys), while postnatal DAPs and DEs levels were associated with delays in development, particularly in motor and social areas among boys [57].

Two more studies evaluated children at three years of age [46,47] with the former finding no association between prenatal maternal levels of TCPy metabolite, although the authors reported an association between postnatal levels of the metabolite TCPy and social development, mainly in boys. Philippat et al. [47], evaluated a population of mothers from California at high risk of having a child with autism (the MARBLES cohort) and found a positive association between exposure to OPs and risk of autism (clinical diagnostic), but only in girls. However, it is necessary to interpret these results with caution since the sample size for girls was very small.

Moreover, a set of four studies longitudinally evaluated outcomes in children aged from one to five years [51], from one to nine years [52,54], and from one to fourteen years of age [59]. Donauer and coworkers [51] did not find any effect at any age on cognition and neurodevelopment in a population from Ohio. However, in another study conducted in a cohort from New York (80% black or Hispanic women) where metabolites of OPs were measured in cord blood samples, the levels of DAPs were negatively associated with mental development at one and two years of age but no association was observed between DAPs and psychomotor development. In this study, certain associations were also found in relation to DAPs and race/ethnicity at one year of age. In this cohort, prenatal levels of DEs were negatively associated with IQ, perceptual reasoning, and working memory in children from seven to nine years, associations that are influenced by PON1 Q192R QQ genotype, which affects CPF metabolism [52]. In a similar vein, and in the same cohort from New York, Furlong et al. [54] found that levels of DEs were associated with poorer social responsiveness in black participants, with a stronger effect found in boys, although in this study only social functioning was evaluated in children from seven to nine years [54]. Similarly, in a study conducted in California (CHAMACOS), maternal DAPs were associated with an increase in autism-related traits in childhood and adolescence, but no association was observed with deficits on specific facial recognition tests for children aged nine and twelve years [59].

Evaluations conducted with prenatally exposed cohorts aged six [61], seven [50], and eight years [58] reported different outcomes. A study conducted in a cohort from the Netherlands found no association between DAPs measured in urine during pregnancy and autism traits [61]. In a Californian cohort (CHAMACOS), prenatal exposure to DAPs was associated with lower cognitive scores, particularly in IQ, verbal comprehension (DAPs), and processing speed (DEs). However, postnatal urinary concentrations of DAPs were not associated with cognitive scores, and in this study, autism traits were not evaluated [50]. In the HOME cohort from Ohio, levels of DAPs were not associated with autism symptoms and no evidence was found to suggest that PON1 polymorphism modified prenatal DAPs exposure or autism risk [58].

Only one study evaluated postnatal exposure, in a cohort of children between the age of six and eleven years from Andalusia (Spain) [55]. In this study, the authors reported that urine DAPs levels of the children were associated with a decrease in verbal comprehension, processing speed, and IQ (primarily in boys). Information about prenatal exposure and the postnatal period until evaluation was estimated according to the proximity of their residence to agricultural areas, with the authors concluding that postnatal exposure to pesticides can negatively affect children’s neuropsychological performance while prenatal exposure was weakly associated with neurodevelopment impairment.

#### 3.4.2. Organochloride Compounds

Only 11 out of 45 studies carried out in humans reported an association between OC pesticides and autism. Of these, nine were classified as VH (81.8%, [63,64,65,66,67,68,69,70,71]), and Kao et al. [68] and Lyall et al. [69] were classified as H-quality. Prenatal exposure was the most frequently studied exposure for OC, except Kao et al. [68] who studied postnatal exposure, while prenatal and postnatal exposure was reported by Kim et al. [65] and Boucher et al. [73].

As previously stated, we evaluated studies of prenatal exposure that measure OCs metabolites in maternal blood during pregnancy, along with breast milk samples of children given postnatal exposure. The literature focuses on DDT and one of its principal breakdown products DDE. Authors that studied the effects of this exposure also assessed other metabolites such as hexachlorocyclohexane (HCH) and hexachlorobenzene (HCB) [63,64,65,66,67,68,69,70,71]. Only two studies assessed other OC compounds (Mirex, [72] and Chlordecone, [73]).

Two studies evaluated children during the first year of life [63,64]. Although no effects were found on reflexes, neurological or psychomotor development after prenatal OC exposure at one month of age [63], a national birth cohort study (Finland) which evaluated children within the age range at risk of ASD (between 0 and 7 years of age) found a link between DDE exposure and an increased likelihood of developing autism [64].

Only one study assessed children’s developmental problems at 18 months of age [73]. Prenatal Chlordecone exposure was measured from umbilical cord blood, whereas postnatal exposure was measured from breast milk collected at 3 months postpartum. Nevertheless, boys prenatally exposed to this OC compound showed poorer motor abilities, whilst no significant association was found between childhood exposure and personal-social, communication, problem-solving, fine and gross motor development [73]. A further evaluation was conducted in children at 4 years of age [72]. While no effects were observed on perceptual-performance, verbal, and motor areas, the INMA cohort showed deficits in cognition, particularly in working memory and quantitative areas (numerical memory or counting and sorting) [72].

Further, four studies evaluated the outcomes in children during the first five years of life, that is, from 13 to 24 months [65], 15 to 38 months [67], 42 to 60 months [71], and four to five years [66]. Even though analysis of maternal OC serum levels did not reveal any specific outcome [65], Jeddy and coworkers [67] reported that an increase of HCB levels was associated with vocabulary comprehension and production deficits in 15-month-old children, while a decrease in intelligibility scores were observed at 38 months of age. The same study found communication problems associated with maternal DDT levels in children aged 38-months-old, but no association was observed between β-HCH or DDE and communication scores in both exposure periods [67]. In addition, a study conducted in Morelos (Mexico) found that DDE exposure during the third trimester of pregnancy was associated with verbal comprehension, cognitive and memory problems [71], while the HOME cohort showed a link between *trans*-nonachlor and an increased risk of developing autism behaviors [66].

Two more studies evaluated children aged between three and 10 years [69] and from four to 9 years [70]. All of these works evaluated the EMA population based on a case-control study that identified biomarkers and their possible association with the risk of developing autism. Lyall and coworkers [69] found no clear evidence that higher levels of DDE and *trans*-nonachlor in maternal serum analyzed during the second trimester of pregnancy increased the risk of the disorder, whilst Hamra et al. [70] found no association between OC exposure and the risk of developing autism.

Postnatal exposure to OC metabolites (measured from breast milk) was only assessed in one study [68] in children aged between 8 and 12 months, finding that exposure to DDT and *trans*-nonachlor were associated with socio-emotional, language, and cognitive deficits, along with motor problems.

#### 3.4.3. Pyrethroid Compounds

With regard to PT, five out of 45 (11.1%) studies were included. Of these, four were classified as VH (80.0%, [74,75,76,77]) and only Watkins et al. [78] was classified as H-quality. All studies assessed prenatal exposure, except for Viel et al. [75,76] who evaluated prenatal and postnatal PT exposure.

The effects of PT exposure were generally assessed by measuring metabolites in maternal urine, cord blood or air samples, and/or child urine. All of these studies assessed PT differently, by 3-phenoxybenzoic acid (3-PBA) (a non-specific metabolite), Permethrin, Cypermethrin and Cyfluthrin (by their *cis* or *trans*-3-(2,2-dichlorovinyl)-2,2-dimethylcyclopropane carboxylic acid (*cis* or *trans*-DCCA) metabolite), 4-Fluoro-3-PBA (4-F-3-PBA) (as a specific metabolite of cyfluthrin) and *cis*-3-(2,2-dibromovinyl)-2,2-DCCA (*cis*-DBCA) (as a deltamethrin specific metabolite) [74,75,76,78]. The only exception was the study by Horton et al. [77], which assessed Permethrin and Piperonyl butoxide (PBO) exposure.

One study evaluated children at two and three years of age [78], while Horton et al. [77] only evaluated children at three years of age. A study conducted in Mexico (the ELEMENT cohort) found lower mental development in 2-year-old children, with stronger effects in girls. The same study found no association between maternal urine 3-PBA levels and motor development at both ages of evaluation [78]. Moreover, studies carried out with a New York cohort (CCEH) also found mental development deficits with PBO air exposure during the third trimester of pregnancy (but not motor problems), whereas no association between Permethrin exposure and mental and motor development were found at two years of age [77]. Another New York cohort study (Mount Sinai Children’s Environmental Health), which measured PT metabolites in maternal urine, assessed outcomes in children aged between one and nine years. The results revealed depression, somatization, behavioral and emotional deficits after 3-PBA exposure, and while *cis*-DCCA was associated with externalizing problems and poorer behavioral and emotional regulation, the *trans*-DCCA isomer was not associated with any adverse effects [74].

Finally, whilst no effects were observed following prenatal PT exposure in the PELAGIE cohort, postnatal 3-PBA and *cis*-DBCA exposure assessed in urine in 6-year-old children was associated with deficits in verbal comprehension and working memory [75], whilst an increased risk of behavioral disorders was also observed following 3-PBA and *trans*-DCCA exposure [76].

#### 3.4.4. Mixtures of Pesticides and Other Potential Neurotoxic Agents

The remaining articles, specifically 12 out of 45 (26.7%), studied the effects of exposure to a common mix of pesticides and other toxicants or several types of pesticides. All of these studies were classified as VH [46,80,81,82,83,84,85,87,88,89,90,91]. Eight out of 12 studies assessed only prenatal exposure, three out of 12 (25.0%, [46,83,88]) assessed both prenatal and postnatal exposure, whereas only one study [87] evaluated postnatal exposure.

Whilst previous studies evaluated a single exposure to pesticides in children, the most common situation in humans is that they are exposed to a wide variety of pesticides, something that is formally taken into account in recent studies related to the concept of exposome science [92]. In this regard, general exposure to pesticides was assessed in three out of 12 studies (25.0%, [82,83,87]). Moreover, six out of 12 studies assessed the effects of exposure to a mix of different pesticides (50.0%, [84,85,88,89,90,91]), whilst the remaining two assessed CM exposure (16.7%, [46,80]), and only Keil et al. [81] assessed NN exposure. With regard to CM, maternal urine, blood, hair, cord blood, or child urine samples were used as measures of prenatal and postnatal exposure to Propoxur, Carbofuranphenol, and Mancozeb [46,80,88]. In addition, prenatal exposure to NN was evaluated by measuring household levels of Imidacloprid [81]. The remainder of the included studies measured prenatal and/or postnatal exposure to OP, PT, or OC, as mentioned in the previous sections.

Two studies evaluated children during the first two years of life [83,84]. A case-control study based on individuals with a primary diagnosis of autism disorder reported an increased risk of ASD following prenatal exposure to pesticides such as CPF, MAL, diazinon, avermectin, and Permethrin during the first year of life [83]. A study carried out in Limpopo (South Africa) found no adverse effects on neurodevelopment at one year of age, whilst DDT exposure was associated with motor problems, and two-year-old children showed communication and language deficits following DDE exposure. This same study assessed exposure to PT metabolite, measured by blood and urine. Socio-emotional problems were observed during the first year of life, while *cis*-DBCA metabolite exposure was linked to communication and language deficit in 1-year-old girls and 2-year-old children of both sexes [84].

Moreover, three studies evaluated outcomes in children aged from 2 to 5 years [87,89,90]. All of these studies evaluated a Californian population (CHARGE cohort), which showed an increased risk of autism in children following prenatal exposure to OP (during the second trimester) and PT (3 months before conception and during the third trimester of pregnancy) [90]. Similarly, Schmidt et al. [89] found the same increased risk of the disorder following OP, PT, and CM exposure, while folic acid (FA) intake decreased this risk. However, no association was found between autism and general postnatal exposure to pesticides [87].

Two more studies evaluated children from one to nine years of age [85] and six to 11 years [82]. One study assessed the Mount Sinai Children’s Environmental Health cohort (New York) and found an association between prenatal urine DMP levels and internalizing problems and better scores in working memory in black children, and whilst DEP was associated with poorer working memory scores, no association was found with PON1 polymorphism [85]. A Denmark population, which evaluated general prenatal pesticide exposure, found an increase in brainstem evoked potential (BAEP) latency in both sexes, as well as impairments in neurobehavioral, language, motor speed, and short-term memory functions, but only in girls [82].

Gunier et al. [91] evaluated 7-year-old children using a number of tests. This study assessed the CHAMACOS cohort, which showed IQ and verbal comprehension deficits following OP, NN, and PT exposure. Furthermore, exposure to NN and PT was also linked to perceptual reasoning problems, while combined exposure to OP and CM was only reported to be associated with IQ deficits [91]. Further, three studies evaluated exposure to only CM at one year [80], two years [88], and three years of age [79]. During the first year of life, a decrease in cognitive abilities in girls, deficits in language and fine motor development in boys, and socio-emotional deficits in both sexes were observed following prenatal exposure to Mancozeb [80]. Moreover, exposure to Propoxur was also associated with motor development problems in boys, while no social behavior deficits were observed [88]. Finally, prenatal urine levels of Carbofuranphenol were linked to a decrease in social and adaptive behaviors, whereas postnatal exposure was associated with language and social behavior deficits [79].

Finally, only Keil et al. [81] assessed prenatal NN exposure using measurements of Imidacloprid in children aged between 3 and 4 years. This study, conducted with a Californian population (CHARGE cohort), found an association between Imidacloprid and autism disorder [81].

## 4. Discussion

### 4.1. Preclinical Studies and ASD

A total of 16 preclinical studies were finally included in the present systematic review. All of them studied some effect on communication or social behavior after being prenatally or postnatally exposed to OP, PT, or GLA.

When dividing the analysis according to developmental stages and compounds, gestational exposure to CPF decreased sociability in mice exposed from GD12-15 to doses from 2 to 5 mg/kg in the best-qualified studies [37,38]. MH classified studies also revealed decreased USV rates and maternal behavior when exposure occurred later during gestation [31,32], but the opposite was also true for both vocalizations and social investigation in male BTBR mice [35] and social interaction rates in wild-type female mice [34]. However, this gestational exposure protocol from GD14 to 17 in BTBR ASD-like mice models was associated with deep alterations in secondary behavioral markers usually observed in ASD patients such as delayed neuromotor development [35], along with an increase in various biomarkers of oxidative stress that are typically associated with autism [36]. Interestingly, this phenomenon of enhanced social and communicative traits was also observed in another model of ASD-like genetic background by using heterozygotes *Reeler* mice and exposing them from GD13 to delivery, as found in Mullen et al. [39], a study categorized as ML quality. This study is of special relevance as the influences of CPO on social outcomes varied depending on sex, where ^+/−^ neonate female mice decreased USVs number whilst their male counterparts increased them, as well as exposed females generally enhanced their social interaction during adolescence. These results support the notion that genetic background and environmental agents interact giving different results in a sex-dimorphic manner. As CPO exposure altered females’ behavior in both ^+/−^ and ^+/−^ conditions, one explanation could be because of the differences that exist between sexes regarding the development of the cholinergic system during early neurodevelopment [93].

Studies of postnatal exposure also yielded inconclusive results. The only H-quality study shows that pre-weaning exposure to CPF altered maternal care and aggressive behavior but enhanced maternal social investigation without affecting the performance of pups in the three-chambers test [30]. Interestingly, one MH-quality study found that pre-weaning exposure to doses as low as 1 mg/kg/day differentially affected the reaction to social novelty in mice depending on the APOE genotype background, with decreased (APOE3) and enhanced (APOE4) rates, both regulated by re-exposure to CPF during adulthood [40]. The earlier data is relevant since isoform 3 is most widely expressed in humans [94]. Finally, the results concerning continuous exposure to CPF [33] or MAL [41] during the whole developmental period (gestational and postnatal ages) showed opposing results characterized by both general enhancement (except for reaction to social novelty in females) and a significant decrease in social traits, respectively. However, we must point out that the differences in quality between these studies (MH vs. ML), the chemical studied (CPF vs. MAL), and the age range of exposure (medium-late gestation to the end of the second postnatal week vs. early gestation to weaning) prevent us from reaching common conclusions. Finally—and in relation to exposure to non-OP compounds—all the three studies included were categorized as MH, and all of them induced an ASD-like phenotype in their mice, using different doses of both GLA [43,44] or CYP [45] during the whole developmental period. Once again, the lack of studies limits the generalizability of these interesting, but insufficient, empirical results.

Based on all this information, we believe that there is not enough empirical support at any developmental stage or exposure protocol to confidently conclude that exposure to OPs or other pesticides can be linked to the development of ASD-like (core) behaviors. However, exposure to CPF during medium gestational ages (around GD12) seems to be the protocol that shows more promise in this regard [37,38]. This hypothesis gains support when using other well-known chemicals that can elicit these behavioral alterations when exposed at this age, as in the case of VPA [95], a drug that has also been linked to the diagnosis of ASD in humans [13]. However, the results provided by Lan and collaborators [37,38] must be replicated in other laboratories and in other rodent species such as rats, which are probably more appropriate models with regard to social outcomes [96], whilst there is also a need to include models with well-known genetic vulnerabilities to study this complex gene/environmental relationship that is thought to underlie ASD.

With respect to this last point, it is noteworthy that only two studies included in the present systematic review used genetic models of ASD (BTBR and heterozygous *Reeler* mice) to study behavioral outcomes [35,39] or molecular biomarkers [36], the two former studies finding enhanced social rates following CPF and CPO exposure. If the current hypothesis—that pesticides are environmental factors that can unmask or worsen the ASD phenotype codified in genes of vulnerability—is valid, these preliminary results point toward another direction, at least in relation to CPF. However, we consider that findings from a sum total of only two studies, which differ markedly, are an insufficient basis upon which to draw firm conclusions. We should not overlook the results obtained by De Felice et al. [35] regarding neuromotor outcomes and later [36] for different molecular markers that are generally linked to ASD.

### 4.2. Clinical Studies and ASD

As reported above, a total of 45 clinical studies were finally included in the present systematic review, 17 of which assessed OP exposure by means of metabolite measures, with two of the 17 studies assessing TCPy as a measure of CPF exposure [46] as well as other OP metabolites [60]. Moreover, 11 out of the 45 studies evaluated OC through metabolite measures, whilst two studied Mirex [72] and Chlordecone exposure [73]. Further, five out of 45 assessed general PT exposure by means of metabolite measures. CM exposure was assessed in two out of 45 (Carbofuranphenol, Zhang et al. [79] and Mancozeb, [80], whereas only Imidacloprid exposure was employed as a measure of NN exposure [81]. Finally, nine out of 45 studies assessed the effects of exposure to a common mix of pesticides.

As reported above, analyses were separated according to pesticide class (OP, OC, PT, CM, NN, and mixtures), period of exposure, and assessment outcome. Considering the studies analyzed assessing OP exposure, 14 out of 17 found effects on cognitive and behavioral functions after either prenatal or postnatal exposure. In particular, studies that assessed the effects of prenatal exposure to OP metabolites at very early ages (first days after delivery) pointed to altered primary reflexes, tone, and behavioral regulation [49,62]. Similarly, VH-quality studies observed alterations in motor function at 5 months [56], whereas H-quality works observed these alterations at 9 months of age [60]. However, these studies lack an adequate follow-up to establish future alterations during childhood or associations with ASD that are more likely to be diagnosed around the age of 18 months and beyond [97]. In this sense, another study classified as VH-quality [48] found associations between prenatal exposure to OPs and alterations in behavioral domains that could influence social relations, along with impaired social scores in boys evaluated at two years of age. Accordingly, social deficits among boys (7–9 years) were also found by Furlong et al. [54] (H-quality), with stronger effects observed in black participants following prenatal exposure, while a study classified as VH [58], with the HOME cohort, did not find any significant associations between OP exposure and autism when their model was adjusted for maternal sociodemographic and perinatal factors (8 years), with the authors indicating that a larger sample size might have allowed them to detect certain associations related to PON1 polymorphisms. Some of the discrepancies in results can be due to differences in OP insecticide exposure in the studied population, given that HOME study enrollment followed the U.S. EPA moves to restrict the residential use of OPs, thus lowering the environmental concentrations in comparison with those to which participants in the Mount Sinai Environmental Health study were exposed [98]. Moreover, another VH-quality study included in this review did not find any association between prenatal exposure to OP and ASD or ADHD [61]. The characteristics of the population included in this study, that is, high socioeconomic level and high levels of exposure mainly through diet (fresh fruit and vegetables) raise an important question regarding the lifestyle and social factors that could increase population resilience to toxicant effects. Further, another study with a long follow-up assessment (from one to 12 years of age) showed childhood and adolescence autism-related traits to be associated with prenatal OP exposure [59]. In addition, an H-quality study [47] found an association between prenatal OP exposure and autism diagnosis at three years of age, but in this case, the effects were only observed in girls. This study is one of the few that uses clinical diagnoses of autism instead of behavioral traits associated with social or communication abilities. Even though the quality of the study is high, the use of a population that is at high risk for ASD may have had some influence on this result. More studies are thus needed to assess OP effects on populations at high risk for ASD.

Moreover, studies categorized as VH-quality also found cognitive and developmental delays in children prenatally exposed to OP at two years of age [53,57] as well as neuropsychological impairments at later ages [50,52].

Regarding early postnatal exposure, VH-quality studies found social impairments among boys at two years of age [57], with similar results being reported by Guo et al. [46] related to CPF exposure at three years of age, whilst Wang et al. [48] reported some changes in the adaptive domain. Moreover, an H-quality study reported neuropsychological impairments associated with postnatal exposures [55].

With regard to OC exposure, 7 out of 11 studies found some cognitive but not social adverse effects in children exposed prenatally or postnatally to this type of pesticide. Two VH-quality studies reported autistic behaviors associated with blood OC metabolite levels [64,66]. All of these use a prenatal exposure protocol, evaluated at early ages (between two and four months of age) [64] and from four to five years of age [66]. In addition, a study using prenatal exposure with a follow-up period between one and five years reported intelligibility, vocabulary comprehension, and production deficits [67], while verbal comprehension, memory, and cognitive problems were observed by Torres-Sánchez et al. [71]. Similarly, cognitive deficits, particularly in working memory and quantitative areas were observed at four years of age following Mirex exposure [72], while Chlordecone exposure was linked to poorer motor ability amongst boys [73]. The same motor and cognitive deficits were also observed in children aged between eight and twelve months following postnatal exposure, in a study categorized as H quality [68]. 

Conversely, the residential uses of PT have been rapidly increasing over the years [99]. In this review, except for the study by Watkins et al. [78] (H-quality), all studies were categorized as VH-quality. Moreover, all the included studies showed behavioral or development deficits, being more evident in the two studies which evaluated postnatal exposure. Prenatal exposure to PT was associated with mental but not motor development problems in children between two and three years of age [77,78]. Children aged up to three years showed behavioral and emotional deficits [74], while the PELAGIE cohort showed an increased risk of behavioral disorders [76], in addition to verbal comprehension and working memory deficits after postnatal exposures [75]. These results are in accordance with those reported by Oulhote and Bouchard [100], who also found behavioral problems in children. In any case, the literature on PTs is scarce, and more longitudinal investigations are needed to verify these findings.

Likewise, all studies that assessed exposure to mixed or different types of pesticides were classified as VH-quality. Moreover, only one study did not find any association with autism symptomology after postnatal mixed exposure. Regarding prenatal exposure to some pesticides mentioned above (CPF, MAL, Diazinon, Avermectin, and Permethrin) appears to be associated with autistic behaviors in children assessed during their first year of life [83]. The same results were observed in the CHARGE cohort exposed to OP and PT [90] in addition to CM and NN [81,89]. Interestingly, Schmidt and coworkers [89] reported a decreased risk of the disorder when mothers received FA supplements during pregnancy. It has previously been reported that FA intake prevents neurodevelopmental and behavioral outcomes such as hyperactivity or verbal deficits, amongst others [101,102]. Accordingly, in a case-control study conducted by the same authors, it was found that gestational FA supplements near the time of conception were associated with a reduction in ASD risk (around 40%) [103]. Moreover, socio-emotional deficits were found after PT exposure at one year of age, as well as communication and language problems among girls, while at two years of age this deficit was observed in both sexes. Similar communicative deficits were observed at two years of age after OC metabolite exposure, followed by motor problems [84]. The same outcomes were observed in girls when they were evaluated between six and 11 years of age, whilst impairments in neurobehavioral and cognitive functions were also observed in this sex [82]. In addition, Furlong et al. [74] found cognitive problems among black children, as well as internalizing deficits after OC exposure. In a similar vein, Gunier et al. [91] found deficits in IQ and comprehension as well as perceptual reasoning problems following OP, PT, and NN exposure, whilst combined OP and CM exposure was only associated with IQ deficits [91]. Prenatal exposure to only carbamates (Mancozeb) at one year of age was associated with cognitive problems among girls, motor and language deficits in boys, and lower socio-emotional scores in both sexes [80]. Further, when studying the effects of exposure at two years of age (Propoxur), Ostrea et al. [88] only found motor problems in boys, whilst at three years of age prenatal and postnatal Carbofuranphenol exposure was found to be associated with social behavior deficits [79].

Taking together, all of this information suggests that there are certain discrepancies in the way in which autism is assessed, with some studies relying on clinical diagnoses of autism whilst others use scale scores to obtain data related to the symptomatology of ASD, which are also shared with other multiple neurodevelopmental disorders such as depression and mood disorders [54]. Moreover, other discrepancies could be related to the substantial variability between studies when assessing the source, route, and period of exposure. Many of the studies assessed exposure through metabolite levels in urine or blood, while others assessed specific compounds such as CPF or MAL. The presence of metabolites in urine may not be exclusively a result of environmental exposure, and in fact, these compounds could be ingested through the diet by humans; therefore, metabolite measures reflect exposure by both environmental and dietary routes [104]. In a similar vein, metabolite evaluation makes it difficult to conclude, because some of these biomarkers are nonspecific (e.g., DAPs or 3-PBA) and instead they arise from a multiple range of compounds with varying levels of toxicity and potency [98]. Further, it is also worth noting that studies conducted in different countries use different compounds and levels of exposure, which could explain some of the discrepancies between results [49,62].

Another challenge is that humans are constantly exposed to a wide variety of pesticides (through diet, house fumigation, or agricultural exposure), but most of the authors reported the effects of exposure to a single pesticide since they studied only a limited number of metabolites. It must also be taken into account that diet could have a pronounced impact on the outcomes observed in children. Diet is a strong variable, and, along with its inherent importance as the source of nutrients and vitamins, it is also associated with socioeconomic status. It remains unclear as to whether the consumption of fresh fruit and vegetables could partially counteract the adverse effects produced by pesticides, or whether there are potential beneficial effects of a diet supplemented with FA [89,103]. Moreover, some studies reported a stronger association between ASD or their symptomatology and sex. Although boys are almost four times more likely than girls to be diagnosed with autism [105], the results did not suggest the existence of a stronger association among boys, since an equal number of studies reported effects among girls. This indicates that environmental factors could equally affect both sexes. Alternatively, other studies find autism-related traits in children of black and unmarried women, thus highlighting the influence of other factors such as diet, vitamin deficiencies, socioeconomic status, or genetics [24].

Another important issue is related to the age at which the outcomes are evaluated since studies assessing behavior at very early stages found more adverse effects than those using a longer follow-up period. It appears that early detection of autism (one or two-week evaluations) can be extremely valuable for detection, prevention, and establishing more effective treatments [106].

### 4.3. Relationship between Preclinical and Clinical Studies Concerning ASD

Comparisons between the preclinical and clinical results summarized in the present systematic review can be made only with regard to OP compound exposure as the remaining families of pesticides have not been systematically studied using animal models. However, the type of compound differs among these studies; since animal studies are more focused on CPF exposure, whilst human studies focus on non-specific OP metabolites. As previously described, preclinical studies have yielded inconsistent results, but exposure during specific gestational ages (around GD12) has the strongest empirical support. Thus, in clinical studies, it is difficult to conclude because of the considerable variability observed in terms of age or type of exposure. Nonetheless, it seems that exposure to OP during prenatal or early postnatal periods is associated with cognitive and social deficits, OC exposure is linked to adverse effects on cognition, and PT exposure is linked to behavioral problems. Therefore, we can conclude that studies conducted in animals included more stable and controlled parameters than those conducted in humans.

## 5. Conclusions

This review provides a comprehensive synthesis of the available evidence from various sources and evaluates the link between exposure to a wide range of pesticides and ASD and associated symptomatology. Our search, however, revealed that OCs have been the most widely studied type of pesticides to date. In particular, the review of preclinical studies highlights that:

- The relation between exposure to different pesticides and the ASD-like phenotype concerning the core symptomatology of autism is relatively under-explored in preclinical research. Even in the case of those compounds for which there is a significant amount of empirical research regarding sociability and/or communicative outcomes (e.g., CPF), the considerable differences between studies regarding exposure protocols (e.g., gestational vs. postnatal or early vs. medium vs. late gestational) make it impossible, in the end, for us to draw any solid conclusions.
- There is a significant gap in the literature as only one study included in the review used rats. Although the relevance of the use of mice is unquestionable, it is known that rat models are closer to humans in terms of genetic background and behavioral regulation, particularly with regard to social behaviors [96].
- Future preclinical research should focus on a more in-depth analysis of exposure to developmental CPF and other pesticides concerning the core (sociability and USVs) and secondary (e.g., neuromotor development) clinical signs of ASD, with a special emphasis on the gestational period around GD12, whilst it will also be necessary to include rat models along with the work carried out with mice.
- The study on wild-type mice should be complemented with the systematic analyses of the interactions of this exposure with the various genetic backgrounds of vulnerability associated with the ASD-like phenotype.

In relation to clinical studies:

- It is difficult to draw solid conclusions as there are a wide variety of studies that differ in many aspects such as route, age, or source of exposure.
- The study of exposure to a single pesticide in humans lacks ecological validity, due to the fact that humans are constantly exposed to a wide range of pesticides through a range of routes such as diet, house fumigation, or agriculture. This wide variability of compounds and environmental exposure could contribute to the heterogeneity of results found in the literature.
- Pesticide exposure appears to co-exist with other factors that may be harmful or beneficial for the development of the nervous system. Examples of other factors that could explain the association between pesticides and ASD are lifestyle, socioeconomic or educational status as well as ethnicity or gender. Moreover, maternal age is an important factor to consider, as the concentration of pesticides in the body increases with age, and so higher maternal ages are more strongly associated with an increased risk of autism in their offspring [107].
- Pesticide exposure did not always show harmful effects when authors considered different covariates, suggesting the existence of certain genetic polymorphisms which could interact with environmental factors and amplify the adverse effects of pesticides in relation to ASD (gene-environment interaction).
- Further clinical research is needed to homogenize exposure in human studies, particularly in terms of exposure to specific pesticides, consideration of other risk factors, as well as the use of a more well-defined follow-up period and validated tools for measuring behavioral outcomes.

## Figures and Tables

**Table 1:** ASD & preclinical studies. From left to right: Study ID, animals’ strain, age at the time of behavioral/physiological assessment, sex analyzed, the xenobiotic agent used, dose, age of exposure, route of exposure, exposure measures of control, behavioral tests, physiological measures, and quality of the study.

Study, Year (Reference)	Strain/Age at Evaluation/Sex	Exposure Agent/Dose/Age/Route	Exposure Control	Behavioral Tests	Behavioral/Pharmacological/Physiological Outcomes	Quality Index
Organophosphate compounds						
Venerosi et al. [30]	Mice/PND > 40. Mums postpartum/Both	CPF. 3 mg/kg/d PND11-15 s.c.	Neuromotor battery	Three-chambers test. Maternal behavior. Nest building. Dark-light test	= sociability and reaction to social novelty. ↓ maternal care. ↓ anxiety in mums. ↓ maternal aggressive behavior. ↑ maternal social investigation. ↓ motricity	H
Lan et al. [37]	Mice/PND5 (maternal care) and PND90 (sociability)/M	CPF. 2.5–5 mg/kg/day GD12-15 Gavage	Weight, reflexes	Maternal behavior. Three-chambers test. Social conditioned place preference. NOR	↓ Sociability. = maternal care. ↓ preference social conditioned place. = NOR	H
Lan et al. [38]	Mice/PND90/Both	CPF. 2.5–5 mg/kg/day GD12-15 Gavage	N.A.	Three-chambers test. Social conditioned place preference.	↓ Social preference males vs. the rest (5mg/kg). ↓ Social preference females vs. males (2.5 mg/kg). = Oxytocin mRNA levels at hypothalamus	H
Venerosi et al. [31]	Mice/PND4-15/Both	CPF. 6 mg/kg/day GD15-18 Gavage	AChE activity, weight, neurobehavioral battery, reflexes	Maternal behavior. USVs. Spontaneous motricity	Altered maternal behavior in CPF exposed mums (increased wall rearing and decreased digging). ↓ USVs (calls/min and duration) only in PND10. ↓ pivoting. ↑ immobility.	MH
Venerosi et al. [32]	Mice/>PND90. Mums postpartum/Both	CPF. 6 mg/kg/day GD15-18 Gavage	N.A.	Maternal aggression. Light-dark test	↓ Maternal aggressive behavior. ↑ Anxiety (Females). = Depressive-like behaviors. Serotonergic hyposensitivity (challenged with fluvoxamine)	MH
De Felice et al. [34]	Mice/>PND70/Both	CPF. 6 mg/kg/day GD14-17 Gavage	Pups, sex ratio, weight	Social Discrimination test	↑ Social investigation (Females). = Reaction to social novelty	MH
Venerosi et al. [33]	Mice/>PND70/Both	CPF. 6 mg/kg/day GD15-PND14 Diet	AChE activity, weight, litter size, sex ratio	Social Recognition test. Open field	↑ Social investigation males (all phases) and females (second exposure to the same partner). ↓ reaction to social novelty (females). ↑ Estrogen Receptor β at Hypothalamus (Males). ↓ Oxytocin at Amygdala (males). ↑ Vasopressin receptor 1a at amygdala. = locomotor activity	MH
De Felice et al. [35]	BTBR Mice/PND4, 6, 8, 8 (USVs). >70 (Sociability and USVs)/Both	CPF. 6 mg/kg/day GD14-17 Gavage	Weight, litter size, sex ratio, mortality, reflexes	USVs. Social Interaction test	↑ (trend) calls. ↑ USVs and social investigation (sniffing) (males to females). Altered developmental neuromotor functioning in exposed mice	MH
De Felice et al. [36]	Mice BTBR/PND1-21/Both	CPF. 6 mg/kg/day GD14-17 Gavage	Weight, litter size	Enzyme immunoassay	↑ 15-F2t-IsoP in BTBR model (vs. wild-type). CPF both reduced (wild-type) and increased (BTBR) 15-F2t-IsoP brain levels in PND1. CPF 15-F2t-IsoP in BTBR animals (males) at PND21. CPF increased PGE2 brain levels in BTBR animals at PND21 (males) and PND70.	MH
Basaure et al. [40]	APOE3 and 4 Mice/PNM5/M	CPF. 1 mg/kg/day PND10-15 Oral 2mg/kg/d PNM5 Diet	Weight	Three chambers test	↑ Sociability in adult exposed (both preweaning exposed and not) APOE3 mice. ↓ reaction to social novelty in APOE3 mice postnatally exposed to CPF. Adult exposure blocked this effect. ↑ reaction to social novelty in APOE4 mice postnatally exposed. Hypothalamus: ↑ Oxytocin mRNA in adult exposed APOE3, ↓ in adult exposed APOE4. Adult exposure increased low expression rates of Vasopressin in APOE3. Adult exposure decreased Vasopressin and vasopressin receptor 1a mRNA levels in APOE4. Adult exposure decreased Estrogen receptor 1, Proopiomelacortina in APOE4, amongst others.	MH
Mullen et al. [39]	Reeler Mice/PND7 (USVs), PND30 (Social interaction)/Both	CPO. 6 mg/mL GD13-Delivery Pump	AChE activity	USVs. Three-chambers test. Open field. MBT	↑ USVs number in exposed ^+/−^Reeler (males) from its vehicle. ↓ USVs number exposed ^+/+^Reeler (males) from its vehicle. ↓ USVs number in exposed females (both genotypes). ↓ USVs duration. ↑ social interaction (sniffing, females, both exposed ^+/+^ and ^+/−^)	ML
Hazarika et al. [42]	Rats/adulthood/Both	PMD. 35 ppm for 30-, 45- and 60-days Adulthood Diet	Weight	Social Interaction test. Open field	= Sociability. ↑ Locomotion (longer exposure protocol). Multiple histopathological disruptions following the different exposure protocols.	ML
Ouardi et al. [41]	Mice/PND21/Both	MAL. 5–15 mg/kg/day GD6-PND21 Gavage	Weight, AChE activity	3-Chambers test. Open field.	↓ Sociability. ↓ Reaction to social novelty. ↑ anxiety (time in periphery, the highest dose). ↑ Brain MDA (PND21). ↓ brain CAT and SOD (PND5-21 for the high exposure, PND21 for the low exposure condition). ↓ brain GST (PND21) and GPX (PND15)	ML
Other families of pesticides						
Laugeray et al. [43]	Mice/PND1-5 (USVs). >PND90 (sociability)/Both (pups) and M (adulthood)	GLA. 0.2–1 mg/kg/3 times per week GD?-PND14 Intranasal	Neurobehavioral battery, weight, reflexes, litter size	Social Interaction test. Three-chambers test. USVs. Plus-maze	↓ USVs in exposed mice (highest dose). ↓ Sociability in the three-chambers test (highest dose). ↓ social interaction with females. = anxiety. ↑ relative gene expression of brain phosphatase and Pten (lowest dose). ↓ relative gene expression of brain phosphatase and Pten and Peg3 genes (highest dose).	MH
Laugeray et al. [45]	Mice/PND1-15 (USVs). >PND90 (sociability)/Both (pups) and M (adulthood)	CYP. 5–20 mg/kg/3 times per week GD6-PND15 Intranasal	Neurobehavioral battery, weight, reflexes, litter size	Social Interaction test. Three-chambers test. USVs. Maternal behavior. Open field. Plus-maze	↓ Maternal behavior (highest dose). ↓ Sociability (lowest dose). = reaction to social novelty (lowest dose). ↓ self-grooming (lowest dose). = USVs. ↑ motricity (velocity in the highest exposed mice). = anxiety (lowest dose). Dysregulation of multiple genes	MH
Dong et al. [44]	Mice/PNW6-10/Both	GLA.12 ug/mL For 8 weeks (mums before mating to delivery) Water	Pregnancy rate, litter size, weight	Social Interaction test. 3-Chambers test. Open field. MBT	↓ Locomotor activity. ↓ Social interaction, sociability, and reaction to social novelty. ↑ compulsivity/anxiety (MBT). ↓ Relative expression of cortical Nrxn1 gene. ↑ Relative abundance of Bacteroidetes bacteria in the gut. ↓ Relative abundance of Firmicutes in the gut. ↓ species diversity in the gut. Gut dysbiosis concerning multiple bacteria at genus level. ↓ Fatty acids biosynthesis.	MH

GD = Gestational day. PND, PNW & PNM = Postnatal day/week/month. s.c. = subcutaneous. F = Female. M = Male. USVs = Ultrasound vocalizations. CPF = Chlorpyrifos. CPO = Chlorpyrifos-Oxon. d = Day. PMD = Phosphomedon. MAL = Malathion. GLA = Glufosinate ammonium. CYP = Pyrethroid Cypermethrin. N.A. = Not applied. AChE = Acetylcholinesterase. NOR = Novel object recognition. MBT = Marble Burying Test. = No effects concerning exposure. ↑ Increased following exposure. **↓** Decreased following exposure. 15-F2t-IsoP = 15-F2t-isoprostane. PGE2 = Prostaglandin E2. APOE = Human Apolipoprotein. MDA = Malondialdehyde. CAT = Catalase. SOD = Superoxide dismutase. GST = Glutathione transferase. GPX = Glutathione peroxidase. Pten = Phosphatase and tensin homolog. Peg3 = Paternally expressed gene 3. H = High quality. MH = Medium-high quality. ML = Medium-low quality.

**Table 2:** ASD & clinical studies. From the left to the right: Study ID, Study design/region, age at evaluation/sex/sample size, type, agent and source of exposure assessment, neurobehavioral or neuropsychological assessment in children, behavioral/physiological outcomes or Diagnostic and quality of the study.

Study, Year (Reference)	Study Design Region	Age at Evaluation/Sex/ Sample Size	Type, Agent, and Source of Exposure Assessment	Neurobehavioral/ Neuropsychological Assessment in Children	Behavioral, Physiological Outcomes/Diagnostic	Quality Index
Organophosphate Compound						
Guo et al. [46]	SMBCS Cohort/Shenyang (China)	3 yo Both/*N* = 377	Env; OP (TCPy) Prenatal (prior to delivery) and postnatal (3 yo) urine samples	Gesell Developmental Schedules	No relationship between prenatal TCPy exposure and neurodevelopment alterations. ↓ Motor and social development related to postnatal exposure mainly in boys	VH
Wang et al. [48]	LWBC Cohort/ Shandong (China)	1–2 yo Both/*N* = 262	B; OP (DAPs) Prenatal (delivery) and postnatal (1 and 2 yo) urine samples	Gesell Developmental Schedules	No association between prenatal or postnatal exposure was found in children at 1 yo. Prenatal exposure to DEs and DAPs was associated with a ↓ in social scores (among boys), while postnatal exposure to DMs and DAPs ↑ adaptive domain in children 2 yo	VH
Zhang et al. [49]	Chinese Cohort/ Shenyang (China)	3 do Both/*N* = 249	Env; OP (DAPs) Prenatal urine samples (prior to delivery)	Neonatal Behavioral Neurological Assessment	↓ Overall neurodevelopment scores after prenatal exposure to OP measured by urine DAPs metabolites. DAPs concentrations, specially DEs was associated with lower scores on the behavior scale and DMs was associated with poorer scores in a passive tone, active tone, and primary reflex	VH
Bouchard et al. [50]	CHAMACOS Cohort/ California (USA)	7 yo Both/*N* = 329	Env; OP (DAPs) Prenatal (13 and 26 gw) and postnatal (6 mo, 1, 2, 3.5, 5 yo) urine samples	Wechsler Intelligence Scale for Children—4th edition	Prenatal DAPs exposure was associated with lower cognitive scores, especially, in IQ, verbal comprehension (DAPs), and processing speed (DEs). Postnatal urinary DAPs concentrations were not associated with cognitive scores	VH
Donauer et al. [51]	HOME Cohort/ Ohio (USA)	Annually from 1 to 5 yo/Both/*N* = 327	Env; OP (DAPs) Prenatal urine samples (16 and 26 gw)	Bayley Scales of Infant Development—2nd edition/Clinical Evaluation of Language Fundamentals—Preschool, 2nd edition/Wechsler Preschool and Primary Scale of Intelligence—3rd edition	No effect on cognitive and neurodevelopmental performance	VH
Engel et al. [52]	Mount Sinai Environmental Health Cohort/New York (USA)	1, 2, and 6-9 yo Both/*N* = 169	Env; OP (DAPs) Maternal blood, cord blood, and prenatal urine samples (between 26 and 28 gw)	Bayley Scales of Infant Development—2nd edition/Wechsler Psychometric Intelligence Test/Wechsler Preschool and Primary Scale of Intelligence—3rd edition/Wechsler Intelligence for Children—4th edition	↓ mental development by DAPs (1 and 2 yo) and DMs (1 yo, race/ethnicity). No association in DAPs and psychomotor development. DEs negatively associated with IQ, perceptual reasoning, and working memory in children 7–9 yo	VH
Eskenazi et al. [53]	CHAMACOS Cohort/ California (USA)	2 yo Both/*N* = 353	Oc; OP (DAPs) Prenatal urine samples (during pregnancy)	Bayley Scales of Infants Development—2nd edition	Maternal DAPs were negatively associated with cognitive and mental abilities as well as with PON1 polymorphism	VH
Kongtip et al. [56]	Cohort/Thailand	5 mo Both/*N* = 50	B; OP (DAPs) Prenatal urine samples (around 28 gw)	Bayley Scales of Infants Development—3rd edition	DEs exposure during 3rd trimester were associated with ↓ in cognitive and motor function, while DAPs maternal levels only affected motor scores	VH
Liu et al. [57]	Chinese Cohort/Shenyang (China)	2 yo Both/*N* = 310	B; OP (DAPs) Prenatal (prior to delivery) and postnatal (2 yo) urine samples	Gesell Developmental Schedules	Prenatal DEs exposure is associated with ↑ risk of being developmentally delayed (in boys). Postnatal DAPs and DEs exposure showed delays in development, especially in motor and social area among boys	VH
Millenson et al. [58]	HOME Cohort/ Ohio (USA)	8 yo Both/*N* = 224	Env; OP (DAPs) Prenatal urine samples (between 13 and 19 gw)	Social Responsiveness Scales	DAPs exposure was not associated with autism symptoms after adjusting for covariates. No evidence that PON1 polymorphism modified prenatal DAPs exposure and autism risk/ASD	VH
Sagiv et al. [59]	CHAMACOS Cohort/ California (USA)	1, 2, 5, 7, 9, 10.5, 12, and 14 yo/Both *N* = 333	Env; OP (DAPs) Prenatal urine samples (13 and 26 wo)	Social Responsiveness Scales/Behavioral Assessment Scales for Children, Version 2/Infant Neuropsychological Evaluation Facial Expression Recognition Test/NEPSY-II Affect Recognition Test	Maternal DAPs were associated with an ↑ in autism-related traits in childhood and adolescence. However, no association was observed on facial recognition test in children 9 and 12 yo/ASD	VH
Van den Dries et al. [61]	Generation R Cohort/ Rotterdam (Netherlands)	6 yo Both/*N* = 622	Env; OP (DAPs) Prenatal (early, mid-, and late pregnancy) and postnatal (6 yo) urine samples	Social Responsiveness Scales	No association between DAPs and autism symptomatology/Autistic traits	VH
Philippat et al. [47]	MARBLES Cohort/ California (USA)	3 yo Both/*N* = 203	Env; OP (DAPs) Prenatal urine samples (1st, 2nd, 3rd trimester)	Autism Diagnostic Observation Schedule/Social Communication Questionnaire/Mullen Scales of Early Learning	OP exposure assessed by DMTP metabolite concentrations tended to ↑ the risk of autism only in girls. No association were observed without sex-stratification/ASD	H
Furlong et al. [54]	Mount Sinai Environmental Health Cohort/New York (USA)	1, 2, 4, 6, 7–9 yo Both/*N* = 136	Env; OP (DAPs) Prenatal urine samples (3rd trimester)	Social Responsiveness Scales	DEs levels were associated with poorer social responsiveness in black participants with a stronger effect on boys. No association was found with DAPs and DMs concentrations /ASD	H
González-Alzaga et al. [55]	Cohort/ Andalusia (Spain)	Between 6 and 11 yo Both/*N* = 256	Env; OP (DAPs) Postnatal urine samples (between 6 and 11 yo)	Wechsler Intelligence Scale for Children—4th edition	DAPs levels associated with a ↓ in verbal comprehension, processing speed, and IQ among boys	H
Silver et al. [60]	Chinese Cohort/ Fuyang (China)	6 wo and 9 mo Both/*N* = 199	Env; OP Prenatal cord blood samples	Peabody Development Motor Scales	No significant findings were observed at 6 wo. Naled and CPF exposure associated with deficits in motor function, among girls at 9 mo	H
Woskie et al. [62]	Cohort/Thailand	Between 0 and 4 do Both/*N* = 82	B; OP (DAPs) Prenatal urine samples (7 gm and prior to delivery)	Brazelton Neonatal Behavioral Assessment Scale	↑ Score in the Range of state cluster score associated with maternal DEP metabolite levels and ↑ urinary DMP metabolite levels was associated with ↑ scores in Habituation cluster	MH
Organochlorine compounds						
Bahena-Medina et al. [63]	Morelos Cohort/Mexico	1 mo Both/*N* = 265	Env; OC Prenatal blood samples (each trimester)	Brazelton Neonatal Behavioral Assessment Scale/Graham—Rosenblatt Scale/Bayley Scales of Infant Development	No effects on reflex, neurological or psychomotor development at 1 mo	VH
Brown et al. [64]	FiPS-A Case-Control/Finland	0–7 yo/Both *N* = 1,556	Env; OC Prenatal blood samples (each trimester)	Autism Diagnostic Interview—Revised	DDE ↑ odds of autism/ASD	VH
Kim et al. [65]	CHECK Cohort/ Seoul, Anyang, Ansan, and Jeju (Korea)	13–24 mo Both/*N* = 140	Env; 38 OC Prenatal blood (pregnancy) and breast milk (30 days after delivery) samples	Bayley Scales of Infant Development—2nd edition	No specific results related to OC pesticides exposure	VH
Puertas et al. [72]	INMA Cohort/ Granada (Spain)	4 yo Both/*N* = 255	Env; OC (Mirex) Placenta samples (at delivery)	McCarthy Scales of Children’s Abilities	↓ Cognitive performance, especially, working memory and quantitative area (numerical memory, counting, and sorting). No effects were observed in perceptual-performance, verbal, and motor areas	VH
Boucher et al. [73]	Timoun Cohort/Guadeloupe	18 mo Both/*N* = 204	Env; OC (Chlordecone) Cord blood and breast milk (3 mo) samples	Ages and Stages Questionnaire/Bayley Scales of Infant Development—2nd edition	Prenatal exposure was associated with poorer motor ability among boys Postnatal exposure: no significant association with personal-social, communication, problem-solving, fine and gross motor scores	VH
Braun et al. [66]	HOME Cohort/ Ohio (USA)	4 and 5 yo Both/*N* = 175	Env; OC Prenatal and blood samples (2nd trimester and at delivery)	Social Responsiveness Scales	Maternal trans-nonachlor ↑ autistic behaviors/ASD	VH
Jeddy et al. [67]	ALSPAC Cohort/England	15–38 mo Girls/*N* = 400	Env; OC Prenatal blood samples (pregnancy)	Adapted versions of the MacArthur Communicative Development Inventory	No association between β-HCH or DDE and communication scores (15 and 38 mo). HCB ↓ vocabulary comprehension and production (15 mo) and ↓ intelligibility scores (38 mo). DDT was associated with a ↓ in communication scores (38 mo)	VH
Hamra et al. [70]	EMA Case-control/California (USA)	4–9 yo Both/*N* = 864	Env; OC Prenatal blood samples (2nd trimester)	Diagnostic and Statistical Manual of Mental Disorder—4th edition	No association between pesticides exposure and odds of autism/ASD	VH
Torres-Sanchez et al. [71]	Morelos Cohort/Mexico	42–60 mo Both/*N* = 203	Env; OC Prenatal blood samples (each trimester)	McCarthy Scales of Children’s Abilities	DDE exposure during the 3rd trimester was associated with ↓ cognition, verbal comprehension, and memory	VH
Kao et al. [68]	FiPS-A Cohort/Taiwan	8–12 mo Both/*N* = 55	Env; 20 OC Postnatal breast milk (between 2 wo and 1 mo) samples	Bayley Scales of Infant Development—3rd edition	DDT and trans-chlordane ↓ cognitive, language, social-emotional, and motor scores	H
Lyall et al. [69]	EMA Case-Control/California (USA)	3–10 yo Both/*N* = 1144	Env; 46 OC Prenatal blood samples (2nd trimester)	Diagnostic and Statistical Manual of Mental Disorders—4th edition, Text Revision	No clear evidence that higher levels of prenatal exposure to p,p’-DDE, and trans-nonachlor increased the risk of ASD/ASD	H
Pyrethroids compounds						
Viel et al. [75]	PELAGIE Cohort/ Brittany (France)	6 yo Both/*N* = 287	Env; PT Prenatal (6–19 gw) and postnatal (6 yo) urine samples	Wechsler Intelligence Scale for Children—4th edition	No effect on neurocognitive scores after prenatal exposure ↓ Verbal comprehension and working memory associated with postnatal exposure to 3-PBA and *cis*-DBCA	VH
Viel et al. [76]	PELAGIE Cohort/ Brittany (France)	6 yo Both/*N* = 287	Env; PT Prenatal (6–19 gw) and postnatal (6 yo) urine samples	Strengths and Difficulties Questionnaire	No significant association between maternal urinary PT metabolites and child neurobehavioral deficits. Childhood urinary levels of 3-PBA and trans-DCCA associated with ↑ odds of behavioral disorders	VH
Furlong et al. [74]	Mount Sinai Children’s Environmental Health Cohort/New York (USA)	1, 2, 4, 6, 7–9 yo Both/*N* = 162	Env; PT Prenatal urine samples (3rd trimester)	Behavior Assessment System for Children/Behavior Rating Inventory of Executive Functioning	3-PBA associated with depression, somatization, behavioral and emotional deficits. Cis-DCCA exposure was associated with behavioral regulation, emotional and externalizing problems, while, trans-DCCA was not associated with adverse effects	VH
Horton et al. [77]	CCEH Cohort/ New York (USA)	3 yo Both/*N* = 342	Env; PT (PBO and Permethrin) Prenatal air (3rd trimester), maternal or cord blood (delivery) samples	Bayley Scales of Infant Development—2nd edition	No association between permethrin air and blood samples with mental or motor development. ↓ in mental development after prenatal PBO exposure, while no association was found in motor development	VH
Watkins et al. [78]	ELEMENT Cohort/Mexico	2–3 yo Both/*N* = 187	Env; PT Prenatal urine samples (3rd trimester)	Bayley Scales for Infant Development—Spanish version, 2nd edition	Lower mental development in 1 yo children, being stronger in girls. No association between maternal 3-PBA and motor development at 2 or 3 years of age	H
Carbamates compounds						
Zhang et al. [79]	SMBCS Cohort/ Shenyang (China)	3 yo Both/*N* = 337	Env; CM (Carbofuranphenol) Prenatal (prior to delivery) and postnatal (3 yo) urine samples	Gesell Developmental Schedules	Prenatal exposure associated with ↓ in social and adaptative behaviors Postnatal exposure associated with language and social behavior deficits	VH
Mora et al. [80]	ISA Cohort/ Matina (Costa Rica)	Pregnancy and 1 yo Both/*N* = 355	B; CM (Mancozeb) Hair, blood, and prenatal urine samples (19, 30, and 33 gw)	Bayley Scales of Infants Development—3rd edition	↓ Cognitive abilities in girls, while language and fine motor development were affected in boys. ↓ social-emotional scores in both sexes	VH
				**Neonicotinoids compounds**		
Keil et al. [81]	CHARGE Case-control/California (USA)	3 and 4 yo Both/*N* = 669	Env; NN (Imidacloprid) Prenatal household by maternal interviews	Autism Diagnostic Interview—Revised/Autism Diagnostic Observation Schedules/Mullen Scales of Early Learning/Vineland Adaptive Behavior Scales/Child Development and Social Communication Questionnaire	Association between autism and Imidacloprid exposure/ASD	VH
Mixture: pesticides and other potential neurotoxic						
Andersen et al. [82]	Cohort/ Denmark	Between 6 and 11 yo Both/*N* = 177	Oc; Insecticides, fungicides, and plant growth regulators Prenatal (1^r^st trimester) exposure No biomonitoring, estimation of exposure	BAEP/Finger Tapping Test/Conner/s Continuous Performance Test II/Wechsler Intelligence Scale for Children—Revised/Woodcock Intelligence Tests of Cognitive Abilities/Copying Test of the Stanford—Binet, 4th edition	↑ Brainstem evoked potential (BAEP) latency (boys and girls). Impairment in neurobehavioral, language, motor speed, and short-term memory functions, only in girls	VH
Gunier et al. [91]	CHAMACOS Cohort/ California (USA)	7 yo Both/*N* = 283	Env; 15 OP, 6 CM, 2 Mn-fungicide, 8 PT, and 1 NN Prenatal house-dust samples	Wechsler Intelligence Scale of Children—4th edition	OP is associated with IQ and verbal comprehension deficits. OP and CM are associated with ↓ IQ. NN, PT, and Mn-fungicides are associated with ↓ in IQ, perceptual reasoning, and verbal comprehension	VH
Schmidt et al. [89]	CHARGE Case-Control/California (USA)	2 and 5 yo Both/*N* = 516	B; OP, PT, and CM Prenatal household (3 mo before conception and during pregnancy)	Autism Diagnostic Observation Schedule/Social Communication Questionnaire/Mullen Scales of Early Learning/Vineland Adaptive Behavior Scales	Exposure to OP, PT, and CM ↑ autism risk, while FA intake ↓ the risk/ASD	VH
Eskenazi et al. [84]	VHEMBRE Cohort/Limpopo (South Africa)	1–2 yo Both/*N* = 705	B; OC and PT Blood and urine (prior and post-delivery) samples	Bayley Scale of Infant Development—3rd edition	(1 yo) No effect of DDT/DDE exposure and neurodevelopment. Cis-DCCA, trans-DCC,A, and 3-PBA were associated with socio-emotional deficits. (2 yo) Motor problems associated with DDT, while DDE ↓ in communication and language. Cis-DBCA were associated with a ↓ in communication and language, among girls (1 yo) and both sexes (2 yo)	VH
Furlong et al. [85]	Mount Sinai Children’s Environmental Health Cohort/New York (USA)	1, 2, 4–7, 9 yo Both/*N* = 404	Env; OP and PT Prenatal (between 25 and 40 gw) urine samples	Behavior Rating Inventory of Executive Functioning/Behavior Assessment System for Children/Wechsler Preschool and Primary Scale of Intelligence—3rd edition/Wechsler Intelligence Scales—4th edition	DMs levels were associated with worse internalizing scores (anxiety scale) and ↑ working memory among black children, while DEs was associated with worse working memory scores. No association was observed with PON1 polymorphism	VH
McCanlies et al. [87]	CHARGE Case-control/California (USA)	2 and 5 yo Both/*N* = 951	Oc; Pesticides Postnatal mother/father interviews	Mullen Scales of Early Learning/Vineland Adaptive Behavior Scales/Autism Diagnostic Observation Schedule/Autism Diagnostic Interview—Revised/Social Communication Questionnaire	No association between pesticides and autism/ASD	VH
Ostrea et al. [88]	Cohort/ Bulacan (Philippines)	2 yo Both/*N* = 754	B; CM (Propoxur) and PT Prenatal maternal blood, hair and postnatal cord blood and children hair	Griffiths Test	Motor development was affected after Propoxur exposure among boys No association was observed between Propoxur exposure and social behavior	VH
Shelton et al. [90]	CHARGE Case-Control/California (USA)	Between 2 and 5 yo Both/*N* = 970	B; OP, CM, PT, and OC Prenatal household (3 mo before conception and during pregnancy)	Autism Diagnostic Observation Schedule/Social Communication Questionnaire/Mullen Scales of Early Learning/Vineland Adaptive Behavior Scales	↑ autism risk after prenatal OP pesticides (1st and 2nd trimester) and PT (3 mo before conception and 3^rt^ trimester)/ASD	VH
Von Ehrenstein et al. [83]	Case-Control/ California (USA)	1 yo/Both *N* = 38,331	Env; Pesticides Prenatal (3 mo before conception and during pregnancy) and postnatal (first year of life) residential samples	Diagnostic and Statistical Manual of Mental Disorders—4th edition, revised	↑ autism risk after prenatal exposure to pesticides such as Glyphosate, CPF, MAL, Diazinon, Avermectin, and Permethrin/ASD	VH

Gw = gestational week. gm, mo = gestational/month old. yo = year old. Oc = occupational. Env = environmental. B = both. DAP = dialkyl phosphate. DM = dimethylphosphate. DE = diethyl phosphate. DMTP = dimethylthiophosphate. DDT = dichlorodiphenyltrichloroethylene. DEP = diethylphosphate. TCPy = 3,5,6-trichloro-2-pyridinol. DDE = dichlorodiphenyldichloroethylene. PON1 = paraxonase 1. DCCA = 3-(2,2-dichlorovinyl)-2,2-dimethylcyclopropane carboxylic acid. DBCA = cis-3-(2,2-dibromovinyl)-2,2-DCCA. 3-PBA = 3-phenoxybenzoic acid. PBO = piperonyl buroxide. HCH = hexachlorocyclohexane. HCB = hexachlorobenzene. Mn = manganese fungicides. IQ = intelligence quotient. FA = folic acid. CPF = Chlorpyrifos. MAL = Malathion. VH = Very high. H = High quality. MH = Medium-high quality. ML = Medium-low quality.

## Data Availability

Not applicable.
